# The chemical biology of IL-12 production *via* the non-canonical NFkB pathway

**DOI:** 10.1039/d0cb00022a

**Published:** 2020-07-22

**Authors:** Peter D. Koch, Mikael J. Pittet, Ralph Weissleder

**Affiliations:** Center for Systems Biology, Massachusetts General Hospital 185 Cambridge St Boston MA 02114 USA rweissleder@mgh.harvard.edu; Department of Systems Biology, Harvard Medical School 200 Longwood Ave Boston MA 02115 USA

## Abstract

Interleukin-12 (IL-12) has emerged as an attractive cytokine for cancer therapy because it has direct anti-cancer effects and additionally plays a critical role in enhancing checkpoint inhibitors. Given these multiple modes of actions, identifying means to pharmacologically induce IL-12 production in the tumor microenvironment has become important. In this review, we highlight therapeutics that promote IL-12 induction in tumor-associated myeloid cells through the non-canonical NFkB pathway. We discuss existing clinical trials and briefly examine the additional pathway targets that warrant further exploration for drug discovery.

## Abstract 2

Interleukin-12 (IL-12) has emerged as an attractive cytokine for cancer therapy because it has direct anti-cancer effects and additionally plays a critical role in enhancing checkpoint inhibitors.

## Graphical abstract

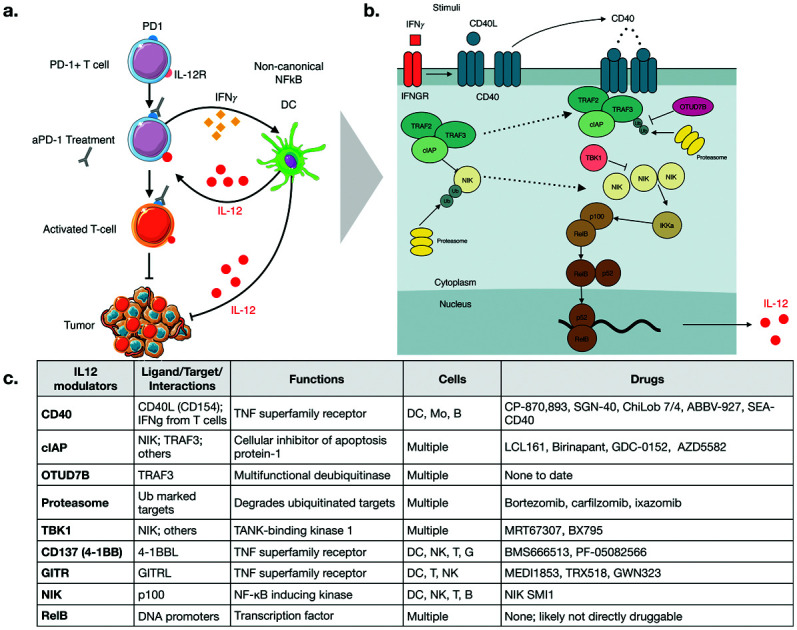

## Introduction

Cancer immunotherapy has fundamentally changed the landscape of oncologic treatment options. Substantial excitement has been generated by the successes of checkpoint inhibitors, which promote the cytotoxic activity of anti-tumorigenic T cells.^1,2^ While such therapies have induced durable remissions in cancers refractory to other treatments, they are not without limitations, most notably in that they work only in a fraction of patients.^3^ As such, numerous avenues are being explored to increase the fraction of responders while minimizing the development of resistance and systemic side effects.

**Fig. 1:**
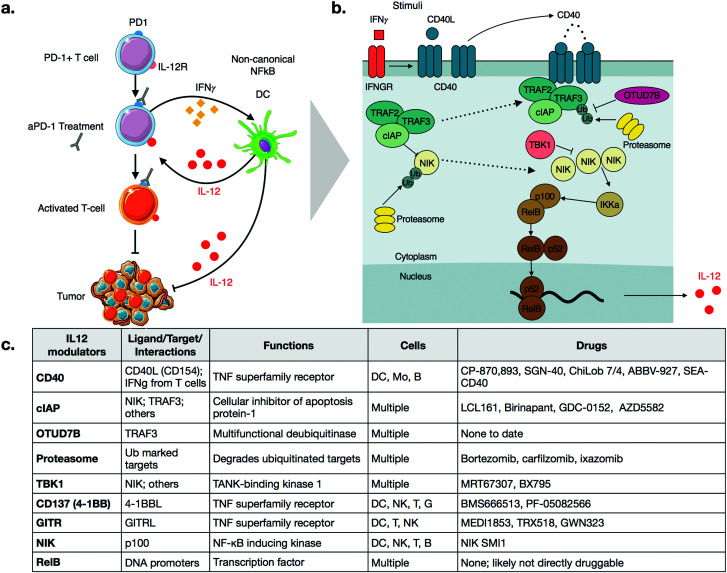
(a) Schematic of IL-12 modulation of checkpoint therapy through T-cell:dendritic cell cross talk. aPD-1 treatment induces IFNγ in T-cells, which promotes production of IL-12 in dendritic cells. IL-12 then further activates anti-tumor T-cells. IL-12 activates other cell types as well, which leads to additional anti. (b) Schematic of the non-canonical NFkB pathway. Activation of CD40, either through CD40L binding or through IFNγ signaling, disrupts the TRAF2-TRAF3-cIAP complex, allowing NIK levels to rise. NIK then promotes p100 processing *via* IKKa, which leads to an active RelB-p52 complex that can translocate to the nucleus and cause transcription of IL-12. (c) Description of various signaling nodes in the non-canonical NFkB pathway.

Similar to targeted therapeutics, a promising approach being pursued is combination therapy.^4–8^ For example, it has been shown in multiple models that activation of innate immune cells can improve the efficacy of checkpoint inhibitors.^9,10^ In particular, several groups have shown a critical role of interleukin-12 (IL-12) in sensitizing tumors to anti-PD-1 therapy.^11–13^ Specifically, production of IL-12 in a subset of tumor-associated dendritic cells, termed DC3, is essential for response to anti-PD-1 treatment.^11,14^ Treatment of mice with anti-PD-1 antibodies leads to an increase in levels of interferon-gamma (IFNγ) in T-cells, which turns on IL-12 production in dendritic cells (Fig. 1a). The full-fledged activation of antitumor immunity triggered by immunotherapy is thus not direct, but rather involves T-cell:dendritic cell crosstalk and is licensed in part by IL-12.^11^

Beyond its emerging role in immunotherapy, IL-12 also has numerous other anti-tumorigenic effects and has thus been of interest therapeutically for some time.^15^ In fact, it is worth noting that one of the oldest cancer immunotherapies, Coley's toxins,^16^ a mixture of dead bacteria, likely induced IL-12 production in patients, as components of bacterial membrane components are known to do so. Today, we know that IL-12 (i) elicits broad anti-tumor effects in multiple cancer models, (ii) acts on various immune cell types including NK-, B-, and T-cells, and (iii) turns on signaling pathways aiding in the activation of T cells.^11,15,17^ Despite these numerous anti-tumorigenic effects, recombinant IL-12 delivered intravenously as a therapeutic has also been shown to have considerable toxicity in humans, thereby limiting its systemic use.^15,17^

Given these findings, it will be important to identify pharmacological interventions that more selectively turn on IL-12 production in tumor-associated immune cells and thus locally broaden the therapeutic window. Preclinical work has demonstrated that IL-12 is produced by multiple immune cell types.^18–21^ However, in tumor microenvironments, the highest IL-12 producing cells have been shown to be dendritic cells in the DC3 cluster.^11^

## The non-canonical NFkB pathway

IL-12 production appears to be tightly regulated by the non-canonical NFkB pathway, as summarized in Fig. 1b.^11^ The central, key regulator of this pathway is NFkB-inducing-kinase (NIK, also known as MAP3K14).^22,23^ In unstimulated cells, NIK levels are very low, as a complex of E3 ligases ubiquitinates the protein. The added chain of ubiquitin has a lysine 48 (K48) linkage, which marks NIK for degradation by the proteasome. This ubiquitin ligase complex that modifies NIK typically consists of the proteins TRAF3, TRAF2, and either cellular Inhibitor of Apoptosis 1 or 2 (cIAP1 or 2). Other proteins may also be included, again depending on contexts such as cellular type. TRAF3 is unable to ubiquitinate directly, so it binds to either cIAP1 or cIAP2 (hereinafter referred to as cIAP), which are responsible for the ubiquitination of NIK.^22^ The bridging of cIAP to TRAF3 is mediated by TRAF2.^24^

Precise triggers of the non-canonical NFkB pathway in dendritic cells are still incompletely known, but dendritic cell sensing of IFNγ may play a role, since dendritic cells in IFNγ receptor-deficient mice show decreased expression of non-canonical NFkB pathway genes.^25^ Other activators of the non-canonical NFkB pathway include ligands of Tumor Necrosis Factor Superfamily Receptors (TNFSFR),^22^ which can be expressed by dendritic cells as well as many other cell types including macrophages, T cells, B cells, NK cells, granuloctyes as well as some non-immune cells.^26^ In dendritic cells, examples of TNFSFRs include CD40, CD137 (4-1BB, TNFRSF9), and the lymphotoxin beta receptor (LTBR).^27^ While the details of signaling vary among the receptors, a common mechanism is that agonization of the receptor by its ligand disrupts cytosolic TRAF3-TRAF2-cIAP complex. Most receptors have a domain that can bind to TRAF3, thereby drawing it away from NIK. With some receptors, cIAP subsequently begins to ubiquitinate TRAF3, marking its degradation.^22,28^ Disruption of the complex frees NIK from ubiquitination, allowing its levels to accumulate slowly, as the gene must be transcribed and then translated. This step is notable because it explains why activation of the non-canonical NFkB pathway is relatively slow, especially in comparison to the canonical NFkB pathway, which elicits a more transient response to stimuli.^29^

As NIK levels increase, it phosphorylates the kinase IKKa, a key kinase relevant in the canonical NFkB pathway as well. IKKa phosphorylates p100, the precursor of NFkB2. Phosphorylation of p100 leads to its ubiquitination and subsequent degradation, forming the active form of NFkB2, p52. NFkB2 p52, in complex, with RelB, then translocate to the nucleus, turning on production of cytokines such as IL-12.^11,30^

As with all innate immune pathways, mechanisms are also in place to prevent inappropriate hyperactivation. The kinase, TBK1, which is typically pro-inflamamatory in other contexts, is inhibitory in non-canonical NFkB pathway, by triggering a signaling cascade to degrade NIK.^31^ OTU domain-containing protein 7B (OTUD7B, also known as Cezanne), is a deubiquitinase which removes ubiquitin from TRAF3, thereby inhibiting its degradation after pathway stimulation.^32^ Going forward, it will be important to identify additional negative regulators of the non-canonical pathways. Most small molecule therapeutics act as inhibitors, so inhibitors of negative regulators have potential as activators of this complex pathway.

It is important to the emphasize that the above description is far from complete, and can vary considerably depending on variables such as cell types and stimuli. There can be crosstalk between and/or mutual activation of the canonical and non-canonical NFkB pathways. Activators of the Toll-like-receptor (TLR), retinoic acid-inducible gene-I-like receptors (RIG-I), and stimulator of interferon genes (STING) receptors, typically associated with canonical NFkB signaling, can also feed into the non-canonical NFkB in certain cell types.^33–36^

## Druggable targets in the non-canonical NFKB pathway

### CD40

CD40 is a TNFSFR expressed by dendritic cells. The naturals ligand for CD40 is CD40L expressed by T-cells.^27^ Binding of CD40 to its ligand triggers the non-canonical NFkB pathway. One notable feature with CD40 signaling is that the CD40L exists as a trimer, and consequently, the binding of CD40L to CD40 is typically marked by oligomerization of CD40 subunits.^37,38^ After engagement, the receptor recruits TRAF3 and signaling occurs as described above. Importantly, while IFNγ does not directly bind CD40, it has been shown to strongly enhances the activation of CD40 signaling, in the context of aPD-1 inhibitors.^11^ Further work may clarify the mechanistic details of this crosstalk.

CP-870,893 (selicrelumab) is an agonistic antibody for CD40, developed by Roche. One of the first phase I trials for CP-870,893, in patients with advanced solid tumors, showed promising results.^39^ Out of twenty-nine patients, four patients with stage IV melanoma showed a partial response, and seven patients with varying tumor types had stable disease. In another trial, CP-870,893 was used in combination with gemcitabine for advanced pancreatic ductal adenocaricnoma.^40^ All patients showed immune stimulation, and four out of twenty two showed a partial response. These patients had hepatic lesions that responded to the drug; biopsies indicated immune infiltration. In both studies, the drug was generally well tolerated, with the most common side effect being cytokine release syndrome. Transient depletion of CD19 + B cells was observed. Whether this effect on B-cell is connected to non-canonical NFkB activation is not entirely clear. However, CD40 is also expressed on B-cells and CD40 therapeutics are also being considered for use in various hematological malignancies.^41^

Several other agonistic antibodies for CD40 are being pursued in both phase 1 and 2 clinical trials (Fig. 1c), including SGN-40 (NCT00079716), SEA-CD40 (NCT02376699), Chi Lob 7/4 (NCT01561911), and ABBV-927 (NCT02988960), often in combination therapy with cytotoxic drugs and/or other immunotherapies.^42–45^ Other biologics such as recombinant CD40Ls are also being considered.^44^ Cellular therapies, such as vaccination with CD40 + dendritic cells or CD40L + T cells are also emerging. BPX101 is a dendritic cell vaccine therapy for metastatic castration-resistant prostate cancer, in which antigen-presenting-cells are collected, and then transduced with an inducible form of CD40.^46^ Specifically, the engineered CD40 receptors have a FKBP12 domain, which allows for the use of a drug, rimiducid, to dimerize the receptor and aid in its activation. The vaccine eliminated tumors in *in vivo* models, and *in vitro* studies confirmed that chemically induced dimerization led to significantly increased levels of IL-12 in dendritic cells. The dimerization allowed for sustained activation of dendritic cells that were resistant to negative feedback. Additionally, inducible dimerization allows for temporal control of CD40 activation. The phase I trial (NCT00868595) of BPX101 had promising results and both anti-tumor and immunostimulatory activities were observed. Further trials are ongoing, and it is plausible to extend this idea to other TNFSFRs, such as CD137 and RANK.^26^

Aside from the above example using rimiducid as a part of a cellular therapy, most approaches targeting CD40 expectedly involve biologics, as it is an extracellular receptor. Most small molecules targeting CD40 signaling are pathway inhibitors being considered for use in inflammatory disease. However, an agonistic peptide mimetic of CD40L has been synthesized using structure aided design. This molecule is trivalent; it has a macrocyclic core, attached to three peptide mimetics that bind CD40.^37^ This molecule had immunostimulatory effects in an infectious disease model,^47^ but has not been pursued for cancer model systems. Nonetheless, it demonstrates that CD40 can be agonized by small molecules.

### cIAP Inhibitors

**Fig. 2:**
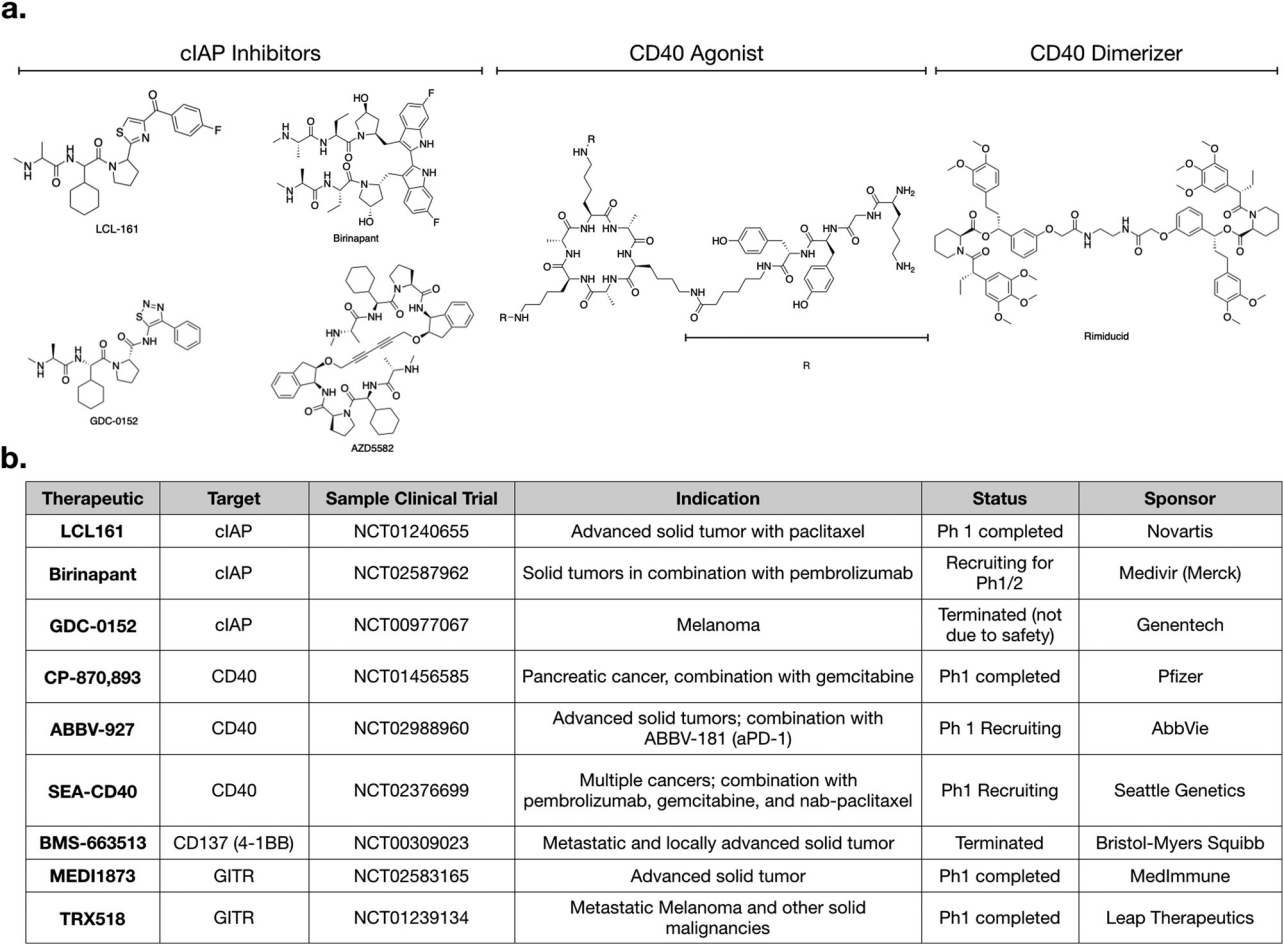
(a) Structure of various cIAP inhibitors (left), CD40 agonist (middle), and rimiducid (right), a tool compound used in cellular therapy. (b) Table of various clinical trials highlighting agents that modulate non-canonical NFkB signaling.

For small molecules, arguably the most promising targets in the non-canonical NFkB pathway for IL-12 production are inhibitors of cellular Inhibitor of Apoptosis 1 and 2 (cIAP1 and cIAP2) (Fig. 1c and 2a). cIAP inhibitors turn on the non-canonical NFkB pathway downstream of TNFSFRs.^48–50^ Numerous cIAP inhibitors have been developed, and several clinical trials have been initiated.

Most cIAP inhibitors are mimetics of second mitochondria-derived activator of caspases (SMAC). SMAC is the endogenous peptide that antagonizes members of the Inhibitor of Apoptosis (IAP) class.^51^ When these SMAC mimetics bind cIAP, they cause cIAP autoubiqitination, which leads to its degradation, consequently allowing NIK levels to increase. The non-canonical NFkB signaling then turns on production of IL-12.

Importantly as the name IAP implies (“inhibitor of apoptosis”), these proteins play an important role in blocking apoptosis. Hence, inhibitors that antagonize IAPs are also pro-apoptotic, and many cIAP inhibitors also have activity against xIAP, another IAP member which is more directly tied to apoptosis without any effect on non-canonical NFkB signaling.^52^ The pathways underlying apoptosis have been reviewed elsewhere,^51,53,54^ but it is important to note that development of various cIAP inhibitors were motivated with this pro-apoptotic mechanism in mind.^55^ IAP members are also often amplified in cancer, furthering the rationale for clinical use of IAP antagonists as a pro-apoptotic agents acting directly on the tumor.^52^

Several preclinical studies have focused on the apoptotic effects of cIAP inhibitors, in both solid tumors and hematological malignancies, often in combination with other cytotoxic drugs.^56–58^ With the realization that cIAP inhibitors also activate non-canonical NFkB signaling, attention has been directed to the immunostimulatory capabilities as well. Most preclinical studies at the *in vivo* level have found broad immunostimulatory effects in multiple cell types.^59^ However, further work has clarified that cIAP inhibitors indeed potently induce IL-12 directly in dendritic cells.^11,60^

As with most small molecules, cIAP inhibitors exhibit polypharmacology, and different drugs have varying degrees of selectivity against cIAP and xIAP. In Table 1, we highlight the affinities of various cIAP inhibitors against selected members of the IAP family. LCL161 and birinapant, two cIAP inhibitors in clinical trials, exhibit preferential activity against cIAP compared to xIAP, but nonetheless are still reasonably potent inhibitors of xIAP. Other inhibitors, such as AZD5582 and GDC-0152, exhibit less preferential activity against cIAP. Whether and how this polypharmacology manifests in a clinical setting remains to be determined.

**Table 1:** IC50 (in nM) of small molecule inhibitors against cIAP1/2 and xIAP. Data taken from multiple ref. 55, 115–117

	cIAP1	cIAP2	xIAP
LCL161	0.4	N/A	35
Birinapant	45	N/A	<1
AZD5582	15	21	15
GDC-0152	17	43	28

It remains to be determined whether the therapeutic mechanism in man of cIAP inhibitors is due to anti-tumor immunity, pro-apoptotic effects, or both. We expect both mechanisms to be relevant. In fact, it is reasonable that they may be synergistic, since promoting apoptosis would lead to dying tumor cells that could be a source of antigens for the immune system.^61^ Conceptually, such a dual mechanism is similar to how increasing numbers of other cytotoxic drugs, such as PARP inhibitors, both kill tumor cells and prime immune cells for activation.^7^ Nonetheless, it is likely that the mechanism is highly dependent on the context of the model system. One study, using the cIAP1 and xIAP inhibitor, LCL161, found that the drug was curative in mouse models of multiple myeloma.^62^ Response was associated with an innate immune signature, and in fact was independent of direct cytotoxic effects of the drug on the tumor.

Numerous clinical trials using cIAP inhibitors have investigated both effects of the drug.^63,64^ Phase 1 studies have established the safety and pharmacokinetic properties of LCL161, developed by Novartis. In one study, a common toxicity was cytokine release syndrome, consistent with immunomodulation.^64^ GDC-0152 is a structurally similar inhibitor,^55^ but its phase 1 trial was terminated for reasons not due to safety or efficacy.

Given that production of IL-12 in dendritic cells enhances aPD-1 therapy in preclinical models,^60^ it will be interesting to examine cIAP inhibitors in combination with checkpoint inhibitors. LCL161 is currently being tested in combination with PDR001 (aPD-1) for multiple myeloma (NCT02890069). Another active trial is testing birinapant, a bivalent SMAC mimetic, in combination with pembrolizumab (aPD-1) in multiple solid tumor types (NCT02587962). Going forward, we can expect to see more trials using cIAP inhibitors. An additional cIAP inhibitor that has not yet been tested clinically, but has shown promising preclinical activity, is AZD5582.^11^ All of these SMAC mimetics have varying biochemical affinities towards the different members of the IAP family, and it will become important to evaluate if any specific target is more important than another, and in what context.

### Other targets and potential probes for non-canonical NFkB modulation

Additional targets for IL-12 production in dendritic cells include other members of the TNFSFR family, such as LTBR, CD137, and GITR.^27,65^ As signaling through these receptors is similar to that of CD40, we refer the reader to more in-depth publications.^66–68^ Most therapeutics targeting these receptors are biologics and Fig. 2b highlights various clinical trials using them.

**Fig. 3:**
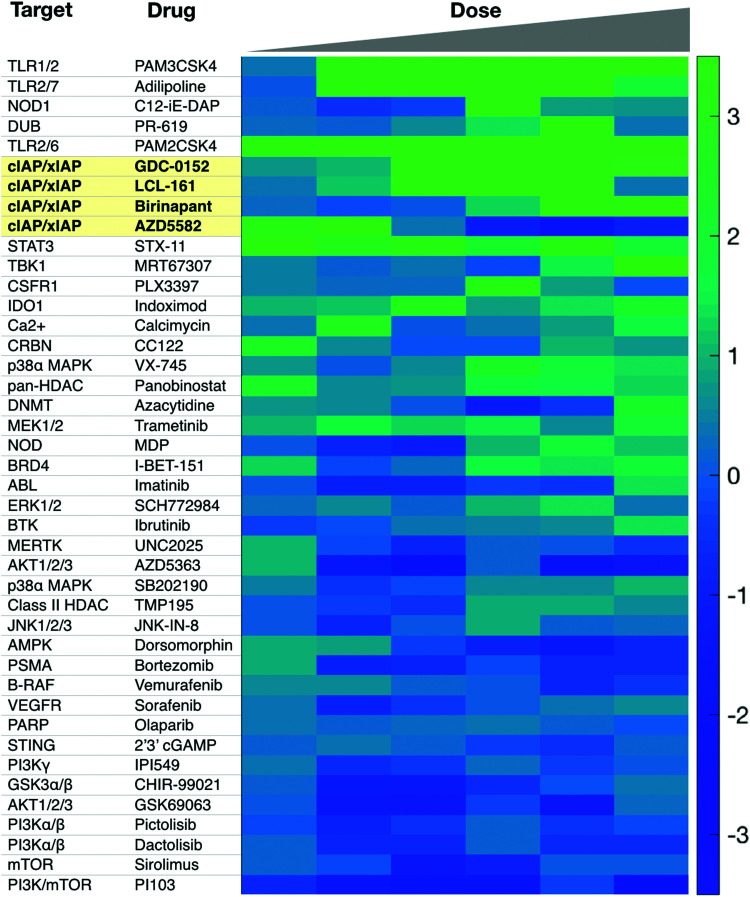
Sample screen for IL-12 inducers in murine dendritic cells. Several cIAP inhibitors scored high in this assay. Reproduced from Koch *et al.* Cell Chem. Biol. (2020).

There is a need for more small molecule modulators of the non-canonical NFkB pathway. Existing small molecule agonists of other innate immune pathways, such as R848, an imidazoquinoline agonist of TLR7/8, or 2′3′ cGAMP, an agonist of STING, enhance IL-12 production but these predominantly signal through canonical NFkB signaling and/or interferon regulatory factor (IRF) pathways.^69,70^ There is some evidence they may also contribute to non-canonical NFkB signaling but more investigation is warranted. Identification of small molecule activators of IL-12 production can be achieved with various screening assays, including using immune cells from an IL-12 YFP reporter mouse^60^ (Fig. 3).

Of the targets shown in Fig. 1, an inhibitor of the deubiquitinase enzyme (DUB), OTUD7B, could specifically activate the non-canonical NFkB pathway. Hu *et al.* showed that depletion of the deubiqitinase enzyme (DUB), OTUD7B, enhanced signaling in dendritic cells.^32^ DUB inhibitors have been developed for cancer therapy.^71^ While none have been developed specifically for OTUD7B, existing chemoproteomic methods could indicate whether any existing inhibitors have significant off target affinity on OTUD7B.^72^ There are only roughly 100 DUBs, so as more inhibitors are developed, an important goal is to characterize their biochemical affinities across the spectrum of DUBs.

Other pathway targets include TRAF3, TBK1, the proteasome, and NIK. TRAF3 is a logical choice from a biological perspective, as it plays a direct role as a pathway inhibitor, and genetic studies clearly indicate that depletion of TRAF3 activates non-canonical NFkB signaling.^73–75^ Unfortunately, there are no specific small molecule probes for TRAF3, and in general, research into inhibitors of E3 ligases is not a well explored space from a chemical perspective. In contrast, TBK1 is druggable, and there are many available inhibitors, such as MRT67307.^76^ However, MRT67307 failed to induce IL-12 production in murine dendritic cells.^60^ Jin *et al.* indicated TBK1 as a negative regulator, but the work was confined to B-cells, so cell type specificity may be an issue.^31^ There are many inhibitors for the proteasome,^77^ including bortezomib, which is FDA approved for multiple indications. It will be reasonable for future work to examine whether bortezomib can modulate non-canonical NFkB signaling. A challenge here is that the proteasome plays multiple roles in non-canonical NFkB signaling, as it degrades both NIK and TRAF3, which have opposing biological roles. Additionally, from a broader perspective, proteasome inhibitors are toxic, and have wide ranging effects on other pathways; these factors might also complicate their use as IL-12 inducers. Lastly, while NIK is druggable, an inhibitor would block, not induce, IL-12 production, and thus lack translational value in cancer immunotherapy. Nonetheless, NIK inhibitors have been developed for treatment of systemic lupus erythematosus,^78^ and these probes may have value as tools to help further dissect pathway biology.

Lastly, it will be interesting to evaluate whether certain small molecule degraders induce IL-12 production. Small molecule degraders are bivalent ligands, composed of an enzyme or receptor inhibitor linked to a E3 ligase binder. The concept behind their mechanism of action is that one part of the molecule binds to its target while another part binds an E3 ligase and brings it near its target; the resulting proximity leads to ubiquitination of the target, thereby promoting its degradation. Numerous studies have indicated that protein degradation has advantages over protein inhibition, and have focused on optimal properties of effective degraders.^79–81^ Interestingly though, a new class of degraders, called SNIPERs,^82^ use SMAC mimetics, such as LCL161, as the E3 ligase binder. Thus, it will be interesting to evaluate whether SNIPERs, by binding cIAP, may also trigger the non-canonical NFkB pathway as an unintended effect. To our knowledge, no study has yet examined whether use of an SMAC mimetic in small molecule degraders might confer immunostimulatory effects. This may give SNIPERs an advantage over other small molecule degraders, such as PROTACs, which use thalidomide analogs instead of SMAC mimetics.

## Canonical NFKB and other signaling pathways

While the primary focus in this review has been on the non-canonical NFkB pathway, it is important to note that IL-12 can also be produced in response to other stimuli as well. Signaling through the canonical NFkB pathway or *via* the interferon-regulatory-factor-3/7 (IRF3/7) pathways can promote IL-12 secretion.^83–85^ Canonical NFkB signaling and IRF3/7 signaling are both activated by upstream pathways. Examples include the Toll-like-receptor (TLR), STING, and RIG-I pathways.^86–88^ These pathways are all similar in that they are triggered by pathogen associated molecular patterns (PAMPs), including bacterial lipopolysaccharide or viral nucleic acid. Each pathway is initiated when a specific receptor binds to a PAMP; activation then funnels into canonical NFkB and/or IRF3/7 signaling, which promote IL-12 secretion.

The canonical NFkB pathway in particular, shares many of the same nodes as the non-canonical pathway. In brief, for the canonical NFkB pathway, an IKK kinase phosphorylates IkB, leading to its degradation by the proteasome. Degradation of the IkB protein allows translocation of a RelA/p50 dimer to the nucleus where it turns on a transcriptional response. In contrast to non-canonical NFkB signaling, canonical pathway responds quickly to stimuli and is transient. For more information on this signaling pathway, we refer the reader elsewhere.^84,85^

Therapeutics acting on the above pathways include PAMPs themselves or synthetic mimetics. For example, the Baccillus Calmette-Guerin (BCG) vaccine, which contains attenuated bacteria, is used in bladder cancer.^89^ Poly-IC, a synthetic dsRNA mimetic that binds RIG-I and TLR3, is being pursued in several cancer immunotherapy strategies,^90,91^ and is also in several clinical trials, including one which it is being used in combination with a CD40 agonist (NCT01008527). Synthetic small molecule agonists have also been developed, as such therapeutics have improved pharmacokinetic properties compared to PAMPs, which are large, and often charged. Imiquimod and resiquimod are imidaquozinolines that binds TLR7 and TLR8.^70,92,93^ There is substantial interest in developing more improved therapeutics on these pathways, which have potential to potently induce IL-12.

## Localized delivery of IL-12 modulators

Ideally, production of IL-12 should be confined to the tumor microenvironment. Systemic administration of recombinant IL-12 has been limited by broad toxicity.^15,17^ A key aspect to improving therapeutic efficacy of IL-12 while minimzing systemic side effects has been to enhance local production in tumor microenevironments. This has and can be achieved by a number of different ways, summarized below.

### Intratumoral delivery

In melanoma, a phase II clinical trial (NCT01502293) is ongoing in which an IL-12 tavokinogene telseplasmid is electroporated into tumors. Preliminary results indicate that intratumoral electroporation enhances antitumor immune responses.^11,17^ This is undoubtedly an impressive technological feat with promising results but may require easily accessible tumors and ideally non-metatstaic lesions, unless these local treatments can produce systemic antitumor immune responses. For biologics, intratumoral injection of CD40 agonists has shown promising efficacy with fewer side effects compared to systemic treatment.^94^

### Targeting myeloid cells with nanotherapeutics

Various nanoformulations have been used to deliver small molecules to the tumor microenvironment. The vast majority of efforts have been centered around the enhanced permeability and retention (EPR) effect and subsequent tumor cell targeting using liposomes, PLGA-PEG, albumin nanoparticles, and graft copolymers, among other formulations.^95–103^ Targeting tumor-associated myeloid cells has also been of interest for diagnostic^104–106^ and therapeutic effects.^107–109^ The nanofromulations most commonly used for myeloid cell targeting include modified dextran and cyclodextrins as well as other carbohydrate-based nanomaterials. Successful examples of myeloid targeting of small molecules include TLR7/8^70,107,110–112^ and cIAP agonists such as LCL161. LCL-161 was complexed to cyclodextrin nanoparticles and shown to (i) increase tumoral IL-12, (ii) decrease tumor volumes, (iii) outperform the free drug control, and (iv) have minimal toxicity.^60^

Further work is needed on clarifying whether certain types of nanoparticles are taken up preferentially by specific types of myeloid or other cells. Advances in intravital imaging and single-cell RNA sequencing will allow for profiling of drug action in various cell types, at a very refined level.^113^ Beyond establishing targeting of myeloid subsets with different nanoformulations,^114^ a major effort will be to incrementally improve drug loading with subset targeting. Overall, we expect nanoformulations to play an important role in enhancing the efficacies of small molecule immunostimulatory drug candidates by directly targeting them to tumor-associated myeloid cells.

## Concluding remarks

It is increasingly clear that local tumoral production of IL-12 is an attractive option for cancer immunotherapy, and has particular promise in synergizing with checkpoint blockade. In this review, we have highlighted the non-canonical NFkB pathway as one source of targets that could be pharmacologically modulated for IL-12 production. At the time of writing, therapeutics targeting this pathway include agonists of TNFSFRs as well inhibitors of cIAP. Clinical trials are ongoing to evaluate these therapeutics but more research is needed to clarify how these drugs work in man. In parallel, it is important to further study non-canonical NFkB signaling to identify new promising approaches, as well as analyze whether any existing drugs have effects on this pathway. Finally, advances in localized delivery of drugs to the tumor microenvironment will be pertinent for IL-12 therapeutics, as it can aid efforts in mitigating immunotoxicity.

## Conflicts of interest

RW is a consultant for Tarveda Pharmaceuticals, ModeRNA, Alivio Therapeutics, Lumicell, Accure Health, and Aikili Biosystems. These commercial relationships are unrelated to the current study. MJP is a consultant for AstraZeneca, Elstar Therapeutics, KSQ Therapeutics, Merck, and Siamab Therapeutics. These commercial relationships are unrelated to the current study. PDK: no relevant disclosures.

## Supplementary Material
